# Development of superhydrophobic and superoleophilic CNT and BNNT coated copper meshes for oil/water separation

**DOI:** 10.1038/s41598-024-65414-5

**Published:** 2024-06-26

**Authors:** Fatemeh Hassani, Abdolreza Aroujalian, Alimorad Rashidi

**Affiliations:** 1 Faculty of Chemical Engineering, Amirkabir University of Technology (Tehran Polytechnic), Tehran, Iran; 2 Nanotechnology Research Center, Research Institute of Petroleum Industry (RIPI), Tehran, Iran

**Keywords:** Multi-walled carbon nanotube coated copper mesh, Boron nitride nanotube coated copper mesh, Oil/water emulsion, Super-hydrophobic membrane, Super-oleophilic membrane, Chemical vapor deposition method, Environmental sciences, Engineering, Nanoscience and technology

## Abstract

In this research, chemical vapor deposition (CVD) method was used to synthesize boron nitride nanotube (BNNT) powder. This method involves heating multi-walled carbon nanotube (MWCNT) and boric acid in the presence of ammonia gas up to 1000 °C. Then MWCNT and synthetic BNNT were coated on the copper mesh via dip-coating method separately to prepare nano-structured membranes for efficient oil/water separation. Various analyzes were performed to identify the synthetic BNNT properties (X-ray diffraction (XRD), Fourier transform infrared spectroscopy (FTIR), Brunauer–Emmett–Teller (BET), field emission scanning electron microscopy (FESEM), energy dispersive spectroscopy (EDS) and prepared coated membranes (FESEM, atomic force microscopy (AFM), water contact angle (WCA), oil contact angle (OCA) and oil/water separation process). Water and oil contact angle analyzes showed the super-oleophilic properties of both membranes with the underwater OCA of about 128°. For the separation process, a dead-end filtration setup was used, and free oil water mixture and o/w emulsion were prepared. So, in the separation process water was retained and decalin passed through both prepared membranes. The flux of CNT coated membrane was about 458 L m^2^ h^−1^, while this amount was 1834 L m^2^ h^−1^ for BNNT coated membrane and 99% separation efficiency was achieved by both of them. This four-fold increase in flux is due to the fact that the inner diameter of boron nitride nanotubes synthesized is four times larger than the inner diameter of MWCNT.

## Introduction

Oil/water separation is a crucial process aiming to mitigate environmental pollution and ensure water quality, because clean water is a fundamental need for our ecosystem and human life^1,2^. Over the years, numerous methods and technologies have been developed for oil/water separation, each with its advantages and limitations. Conventional techniques such as centrifugation^3^, gravity settling^4^, gas flotation^5^, coagulation^6,7^ all operate based on gravity differences are not effective for separating oil/water emulsions because of tiny size of oil droplets^8^. Membrane technology is widely known as a major process for water purification because of low capital and operating cost, low energy consumption, easy operation and high efficiency^2^. But membrane technologies also have some drawbacks and a trade-off between flux and rejection is known as the most important one^2^. Using nanomaterials can overcome this problem.

Carbon nanotubes (CNTs) which contains single-walled carbon nanotubes (SWCNTs) and multi-walled carbon nanotubes (MWCNTs) are one of the most attracted nanomaterials used in this technology, because of high specific surface area, high mechanical strength, excellent chemical inertness and encouraging adsorption capacity^2^. For example, Zhao et al. reported a superhydrophobic poly vinylidene fluoride hexafluoropropylene, carbon nanotubes membrane with highly porous structures for oil–water separation^9^. In the other work, Gu et al. fabricated Janus polymer/carbon nanotube (CNT) hybrid membranes for oil/water separation^10^ and Saththasivam et al. presented a new concept to engineer CNT membranes with a three-dimensional architecture towards fast and efficient oil/water separation^8^.

Boron nitride nanotubes (BNNTs), an analogous form of CNT, have similar structure with CNTs and also possess high thermal conductivity, good mechanical performance, outstanding electrical insulation, as well as excellent chemical stability and superb oxidation resistance^11^.

Both CNTs and BNNTs have great mechanical and thermal resistance but CNTs oxidize in air at 400–600°C and burn totally at 700 °C, therefore their applications limited in high temperature^12,13^. Unlike CNTs, BNNTs are polar nanoparticles because of partial atomic charge on boron and nitrogen atoms and covalent bonds between B and N atoms^14,15^. In contrast to CNTs, a few literatures are available on BNNT separation applications due to the low yield of its synthesis reaction.

For the synthesis of BNNT, a number of studies have been done using different techniques. In the arc-discharge technique, the produced BNNTs had a large amount of metal catalytic nanoparticles that created a gray color in the product^16^. It was observed bamboo-like structures in the BNNTs synthesized by ball milling method, also some B/B-N reactants remained as an impurity that was difficult to remove^17–21^. using triple DC thermal plasma reactor with hydrogen injection made highly BNNTs having multi-walls (≤ 5 walls) and a small diameter (~ 7 nm) but a simple set-up and equipment was not accomplished^22^. Although the BNNTs produced by BOCVD (boron oxide CVD) method was highly pure but the flow of NH_3_ gas was very high in this method which was not safe^23^. For the synthesis of single-walled BNNTs, the laser vaporization method could be the most effective approach. However, the lengths of the produced BNNTs by this method are only hundreds of nanometers^24^. Chemical vapor deposition (CVD) method as the most promising way used to BNNT production^25,26^, because this method has relatively simple procedure and recommended for the production of multi-walled BNNT in large-scale at high yield and purity^2,23^. In the present study, a simple CVD method is used to make BNNT following procedure (ii) of Deepak’s work^27^.

In the other research, CNT hybrid membranes with other polymers (poly vinylidene fluoride-hexafluoropropylene/CNT^9^ and Janus polymer/CNT^10^) and composite membranes (CNT/TiO_2_^28^ and CNT/polysulfone^29^ were used for oil/water separation but in this study, CNT and BNNT was coated on the copper mesh individually for high efficient oil/water separation with higher flux. Achieving to higher fluxes using CNT and BNNT coated copper mesh is due to the frictionless properties of these materials^30^.

In this study, after BNNT synthesis by CVD technique, MWCNT and BNNT coated copper meshes named C_1_ and B_1_ were prepared and their ability to separation of oil/water mixture and emulsion was examined and comprised. CNT and BNNT show quiet different wettability properties and this characteristic was investigated by measuring water contact angle (WCA), oil contact angle (OCA) and underwater oil contact angle (UOCA) of the prepared membranes.

## Materials and methods

### Materials

For the BNNT synthesis, MWCNT powder (with the diameter of 30–50 nm, the length of 5 µm and the surface area of 139.3 m^2^/g) was supplied from Research Institute of Petroleum Industry (RIPI). Boric acid (B(OH)_3_) was prepared by Sigma Aldrich Co.

Copper mesh no. 120 with pore size of 125 microns as substrate, was bought from a local company. Thermoplastic polyurethane (TPU) with a density of 1.12 g/cm3 as a binder, N, N–Dimethylformamide (DMF, 99.5%, Aladin) as a solvent, Sodium dodecyl sulfate (SDS, CH_3_(CH_2_)_11_OSO_3_Na) as a surfactant, Tween 80 (Polysorbate 80, C_64_H_126_O_26_) as an emulsifier, Ethanol (purity of 96%) were purchased from the Polymer Research Institute in Iran.

### Synthesis of boron nitride nanotubes

**Figure 1:**
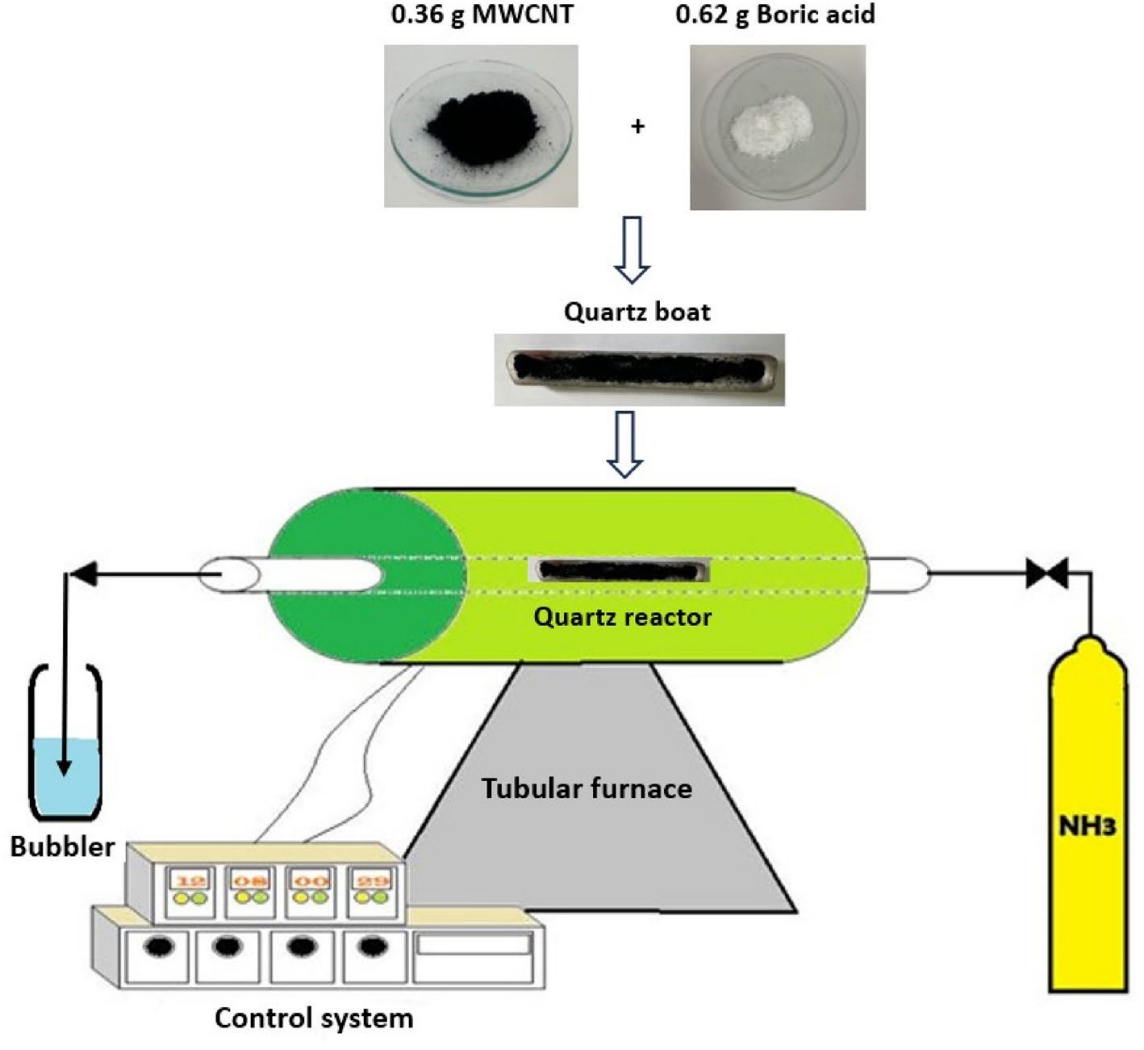
Schematic diagram of BNNT synthesis by CVD method (reactor temperature = 200 °C for 2h, 1000 °C for 3h).

In order to synthesis boron nitride nanotubes, The following procedure as CVD method was employe^27^. According to schematic diagram of BNNT synthesis which is shown in Fig. 1, a mixture of MWCNT (0.36 gr) and boric acid (0.62 gr) was poured into a quartz tube which was horizontally placed inside a larger quartz reactor. This system was placed inside a tubular furnace which ammonia gas with the flow rate of 12 sccm passed through the quartz tube, the furnace temperature was initially set at 200 °C for 2 h and then slowly increased to 1000 °C at a rate of 5°/min and remained at this temperature for 3 h. After that, the system was allowed to cool at the same rate to reach the ambient temperature. Finally, the light gray powders mainly seen at the bottom of the inner tube were known as BNNT powders.

### Preparation of CNT and BNNT membranes

Two separate coating solutions of CNT and BNNT powders in DMF solvent were made using TPU as binder and SDS as surfactant. At first, a 4 wt.% TPU in DMF solution was prepared under stirring for 3 h to have a homogenous solution. Next, 0.92 g of CNT and 0.4 g of SDS were dispersed in the solution using bath sonication for 6 h. This process was repeated for BNNT as well to have CNT and BNNT stable and homogenous coating solution separately.

**Figure 2:**
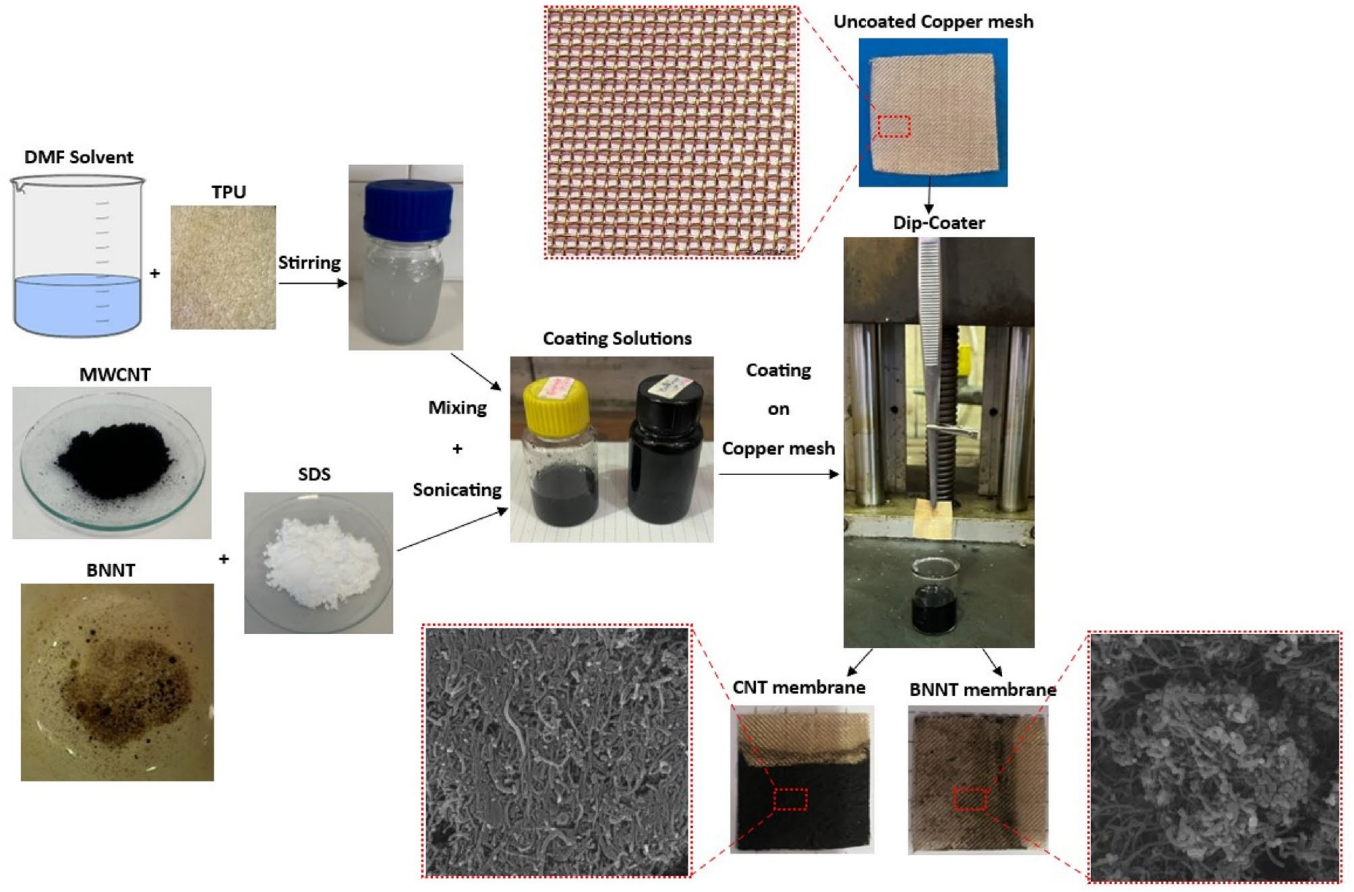
Schematic illustration of preparation of CNT and BNNT coated copper mesh (TPU in DMF solution: 4 wt.%, nanoparticles: 0.92 g, SDS: 0.4 g, dip coater speed: 3 cm/min).

Copper mesh pieces (2cm * 2cm) were first washed by detergent and distilled water well, then placed into the ethanol under bath sonication for 15 min to remove any pollution. The prepared CNT and BNNT coating solutions were coated on the clean copper mesh using a dip coater with the lowest speed (3 cm/min). Eventually, the coated meshes were air-dried well to completely remove the solvent and named C_1_ (CNT coated) and B_1_ (BNNT coated) in this study. Schematic illustration of preparation of CNT and BNNT coated copper mesh is shown in Fig. 2.

### Free oil water mixture and oil/water emulsion preparation

Free oil water mixture was prepared by mixing equal volumes of water and decalin which marked in red dye to distinguish the separation better. For the preparation of oil in water emulsion, distilled water was used as the aqueous phase, decalin was used as the oil and dispersed phase, and tween 80 was used as an emulsifier for emulsion stability. Here, 50 wt.% O/W emulsion is used. For this purpose, 20 ml of decalin with Tween 80 (2 wt.%) as an emulsifier were placed on the stirrer for 15 min. After that, 20 ml of water was added drop by drop to the solution and the mixture was stirred for 5 h at 2500 rpm to form a stable O/W emulsion.

### Oil/water separation by coated meshes

**Figure 3:**
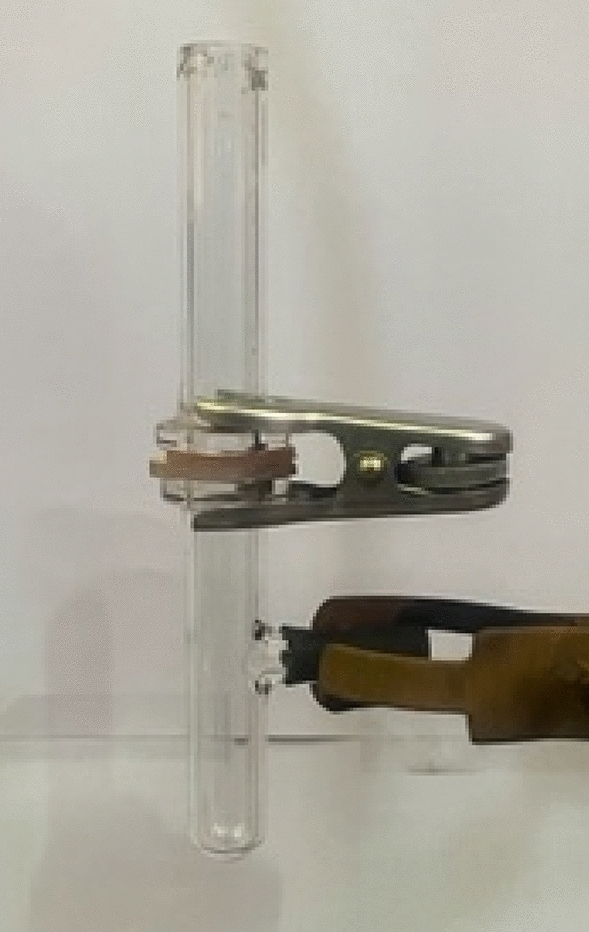
The oil/water separation set-up.

The separation set-up prepared to investigate the coated membranes performance can be seen in the Fig. 3 which includes two glass tubes with a diameter of 25 mm. The coated membrane is fixed between these two glasses and the solution is poured on the membrane.

### Characterization

To identify the synthetic BNNT powder and compare it with CNT, various analyzes were performed. Field emission scanning electron microscopy (FESEM, MIRA3TESCAN-XMU model) to investigate the morphology and nanotube pore diameter, energy dispersive spectroscopy (EDS) to specified elemental composition, Fourier transform infrared spectroscopy (FTIR, Bruker Tensor 27 model) to identify functional groups. Brunauer, Emmett and Teller method (BET) to obtain the specific surface area and the pore diameter. X-ray diffraction (XRD, Intel EQUINOX3000 model) to inquire to the phase structure and crystallinity of the powder were employed.

After coating process, the CNT and BNNT coated membranes were examined by field emission scanning electron microscopy (FESEM). Also, the wettability behavior of coated membranes was determined by water contact angle (WCA), oil contact angle (OCA) and underwater oil contact angle (UOCA) tests with CAG-20 Jikan apparatus. To evaluate topographic characteristics of the prepared membranes such as surface roughness, atomic force microscope (AFM, Bruker, ICON model) analyze in direct contact mode was carried out.

Finally, to calculate the flux as an important factor for evaluating prepared membranes, Eq. (1) ^31^ was used. Then, to determine the ability of CNT and BNNT coated meshes to oil/water separation, Eq. (2) ^32^ was applied:

1 $$ F = \frac{V}{A.t} $$

where V is the volume of permeated liquid, A indicates the effective membrane area and t is the time spent.

2 $$ R \left( \% \right) = \left( {1 - \frac{{C_{P} }}{{C_{f} }}} \right)*100 $$

where $C_{P}$ and $C_{f}$ demonstrate permeate concentration and feed concentration respectively.

## Results and discussion

### BNNT characterizations results

The results of specific surface area, pore volume and average pore diameter of both CNT and BNNT are presented in Table 1. The higher level of CNT’s specific surface area (139 m^2^ g^−1^) and pore volume (0.84 cm^3^ g^−1^) compared to BNNT (51.6 m^2^ g^−1^, 0.25 cm^3^ g^−1^) in these measurements is due to the high purity of the CNT powder used in this study. The impurities existing in the synthesized BNNT have a small level of surface area^33^ which caused the lower BET surface area in comparison to pure CNT.

**Table 1:** BET analysis results for CNT and BNNT powder.

Sample name	BET specific surface area (m^2^ g^−1^)	Pore volume (cm^3^ g^−1^)	Average pore diameter (nm)
CNT	139.3	8.441E − 01	45
BNNT	51.6	2.559E − 01	120

**Figure 4:**
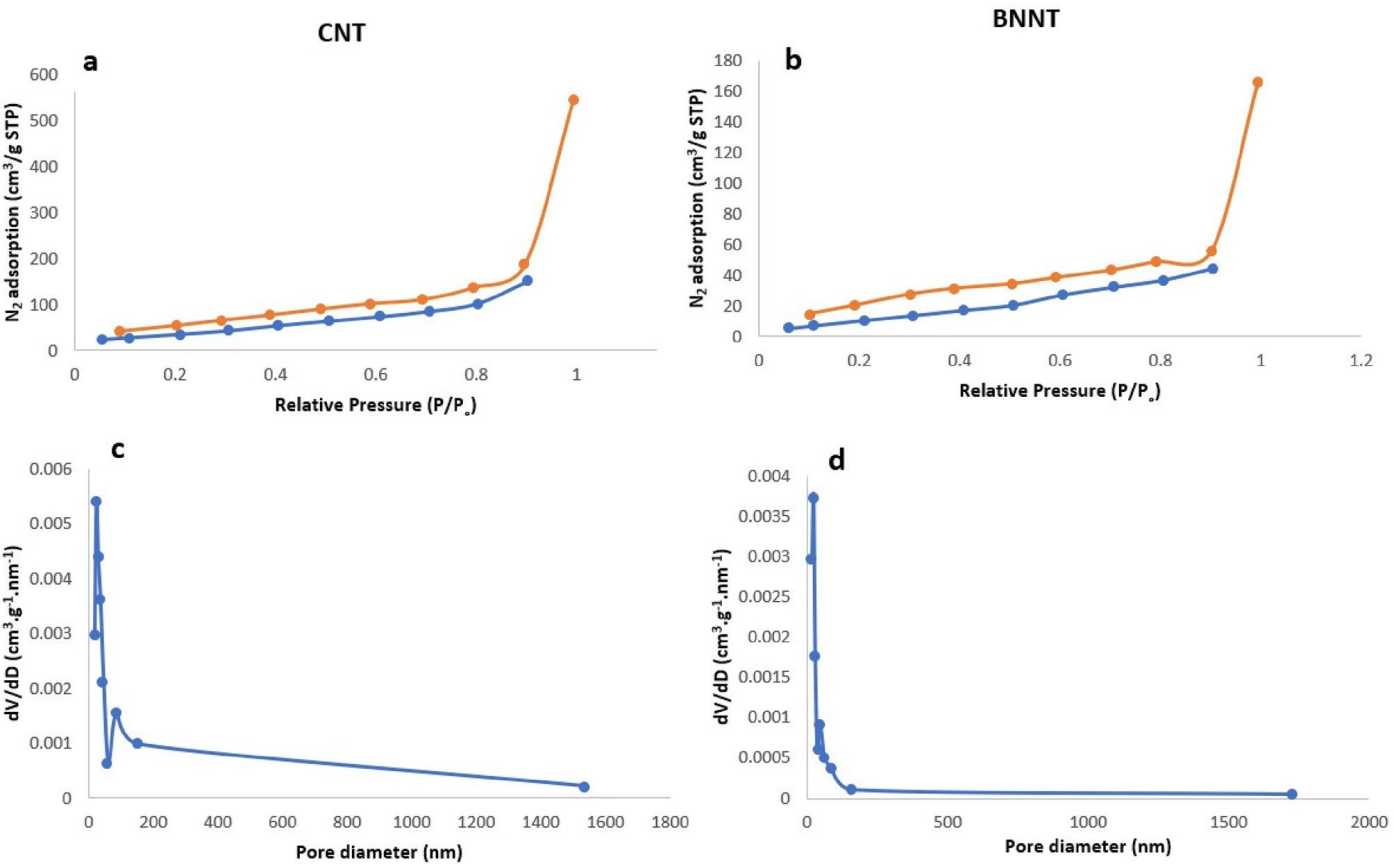
(**a**) The nitrogen adsorption–desorption isotherm of CNT and (**b**) BNNT synthesis powder. (**c**) Pore size distribution of CNT and (**d**) BNNT synthesis powder (BET analyze).

Figure 4a,b shows the nitrogen adsorption–desorption isotherms for CNT and BNNT respectively. Also, BJH method was used to determine the pore diameter of CNT and BNNT (Fig. 4c,d). It can be found that both CNT and BNNT samples showed a typical type III isotherm according to IUPAC (International Union of Pure and Applied Chemistry) classification. Because the adsorbate (N_2_) uptake increases exponentially with increasing relative pressure as shown in Fig. 4a,b. According to the IUPAC division scheme, hysteresis loops are classified into four types (type H1∼H4)^34^. The type-III hysteresis loop confirms the existence of the mesopores and macropores in the samples^35^. Materials that give rise to H3 hysteresis have slit-shaped pores (the isotherms revealing type H3 do not show any limiting adsorption at high P/P_0_). The amount of nitrogen adsorbed by CNT powder was reached to about 600 cm^3^ g^−1^, while this amount was reached to 170 cm^3^ g^−1^ by synthesized BNNT. More porous structure and more internal adsorption sites inside the pure CNT compared to unpure BNNT has caused the higher adsorbed nitrogen amount.

**Figure 5:**
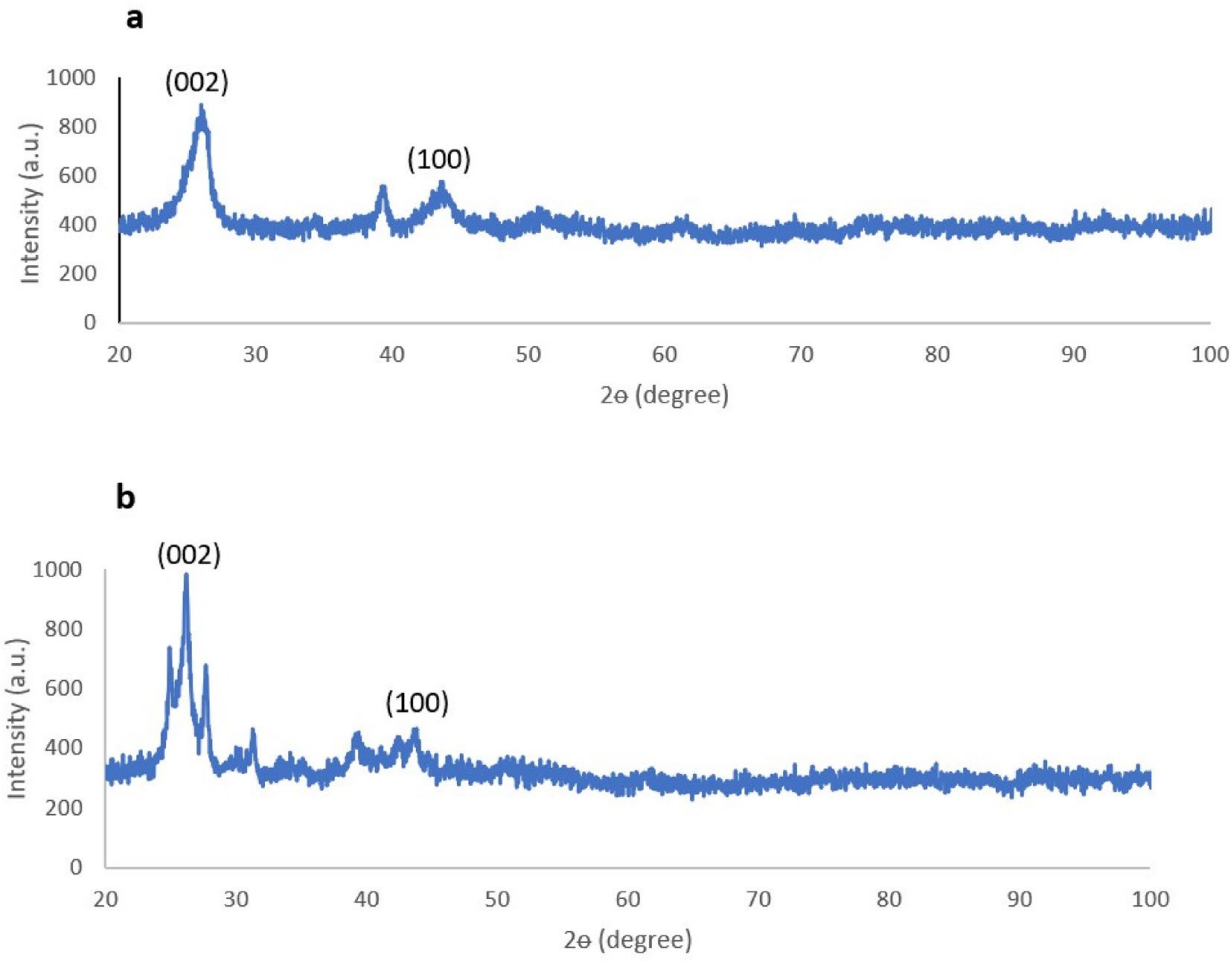
XRD pattern of (**a**) CNT and (**b**) BNNT powder.

To determine the crystalline structural properties of CNT and BNNT powders, X-ray diffraction was carried out and the results of XRD analysis are shown in Fig. 5. However CNT and BNNT are classified as crystalline materials, distinct XRD peaks showed in the Fig. 5a,b are related to their periodic structure^36,37^.

As it is clearly found from the figure, both CNT and BBNT had (002) peak at 26° (2θ) and (100) peak at 42° (2θ).

It can be considered the carbon atoms in CNTs as 3D optical diffractors scattering light at different specific angles. So from the position and intensity of diffracted beams, information on aligning graphene sheets of CNT can be extracted^37,38^. Therefore, it is possible to determine the alignment degrees of nanotubes by measuring the intensity of (002) diffraction. Figure 5 shows that the intensity of (002) peak in CNT was about 850 which is lower than this value in BBNT (1000), because CNTs are better aligned than BNNTs^39^.

X-ray diffraction XRD pattern of the BNNT exhibiting two main diffraction peaks with d-spacing of 0.336 and 0.215 nm are identified to be interlayer distances of the (002) and (100) planes of the hexagonal boron nitride (h-BN) structure.

**Figure 6:**
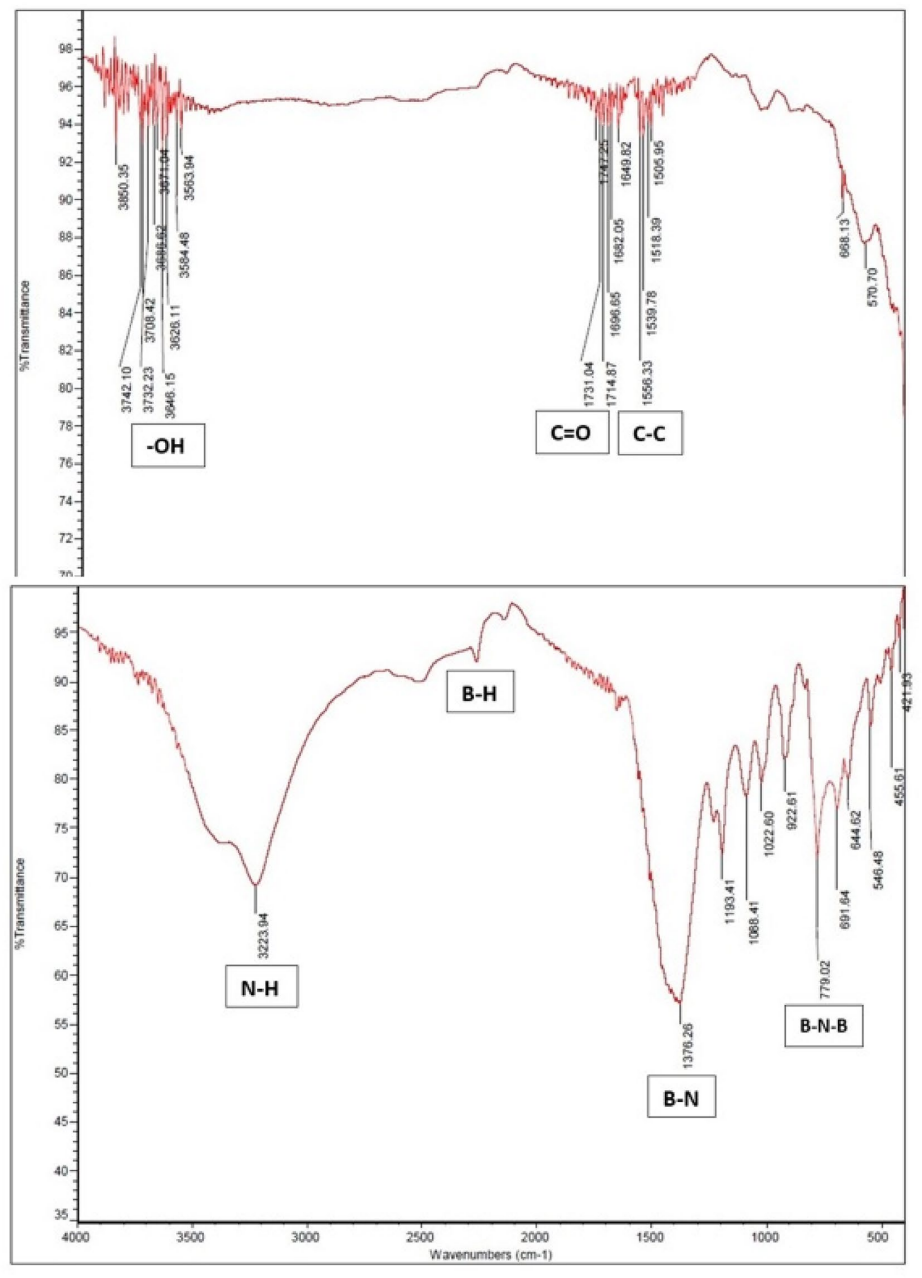
FTIR spectra of (**a**) CNT and (**b**) BNNT samples.

Figure 6a,b shows the FTIR spectra of CNT and prepared BNNT powder. The peaks at the 3700 cm^−1^, 1700 cm^−1^ and 1500 cm^−1^ regions refers to –OH, C=O and C–C functional groups respectively which existed in CNT. Also, this spectra FTIR is similar to the standard CNT FTIR spectra^40–42^ in the Fig. 6b, it can be seen some different peaks at 3200 cm^−1^, 2200 cm^−1^, 1300 cm^−1^, 770 cm^−1^ regions indicated N–H, B–H, B–N, B–N–B functional groups respectively which are the confirmation of the correct boron nitride nanotubes synthesis^43^.

**Figure 7:**
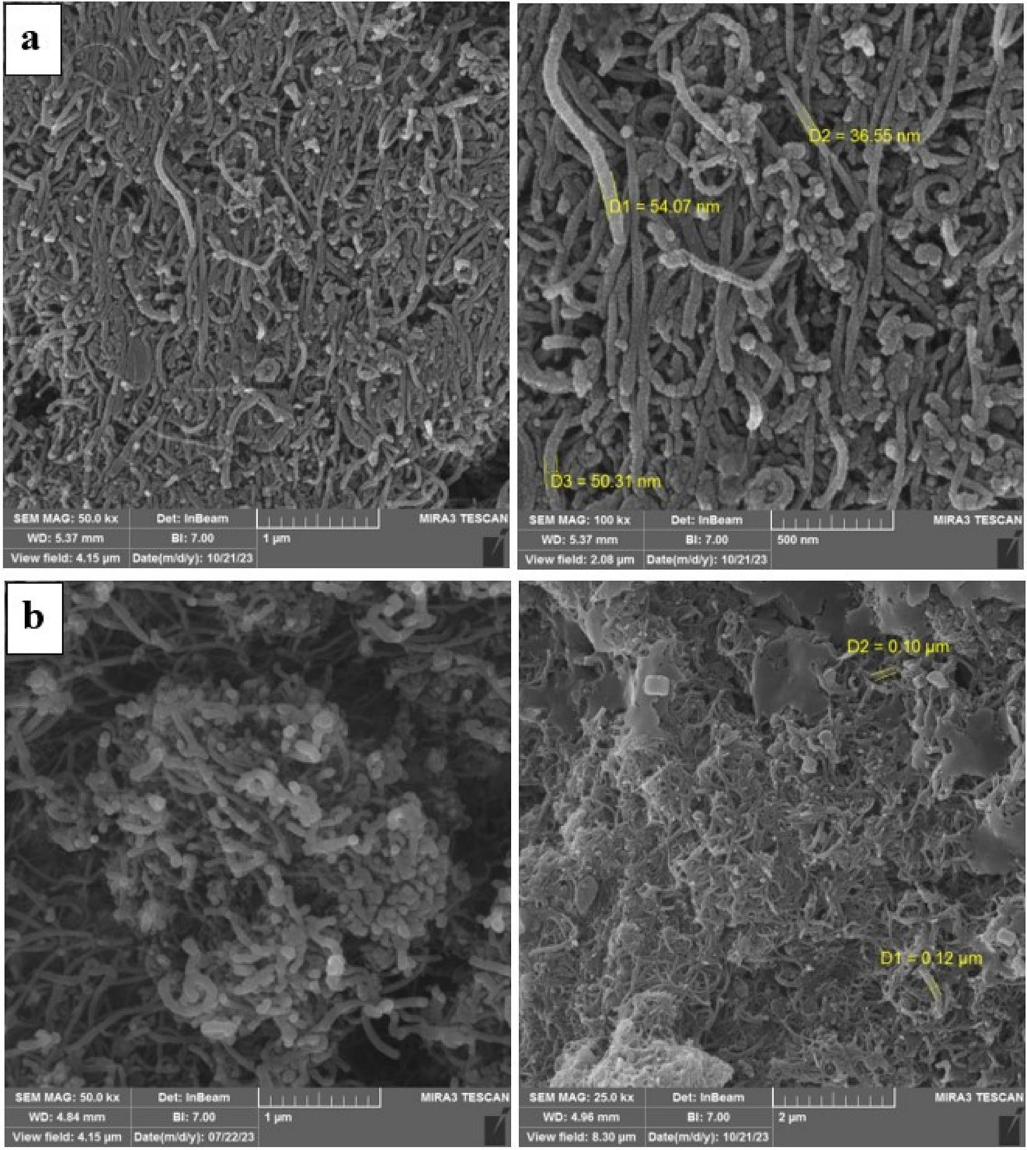
FESEM images of (**a**) CNT powder and (**b**) BNNT powder synthesized.

Figure 7 shows the morphology of CNT and prepared BNNT samples, there was no significant morphology difference between them and the nanotube structure of both is visible^44^. The average pore diameter of employed CNT in this research was about 45 nm and the length of it was around 5 µm. The prepared BNNT had the pore diameter of 0.12 µ and the length of about 3 µ. So, the pore diameter of BNNT powder was three times larger than CNT powder.

**Figure 8:**
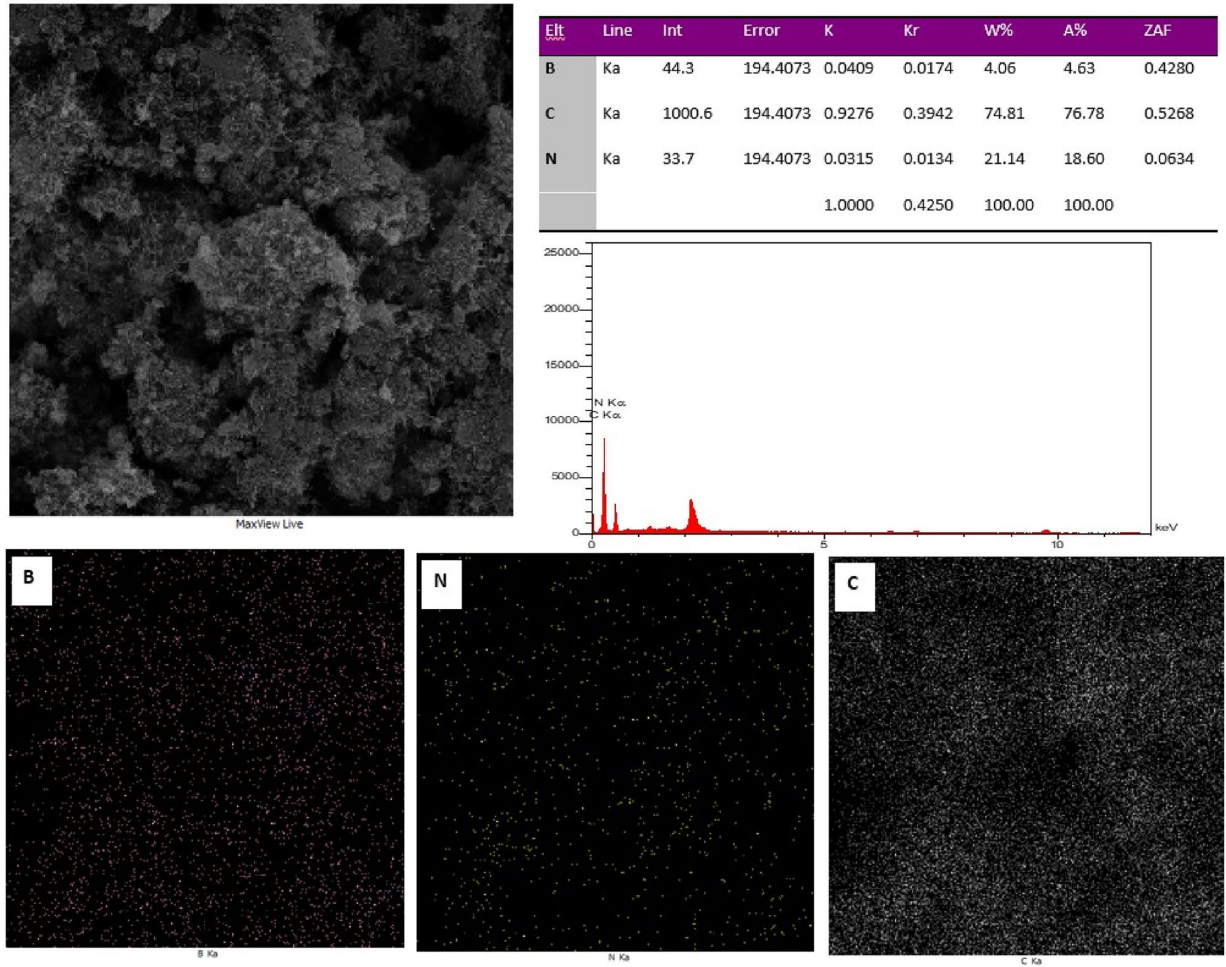
EDS spectra and elemental mapping of B, N and C in the synthetic BNNT.

Figure 8 shows the EDS spectra and elemental mapping of synthetic BNNT to illustrate the chemical composition of prepared sample. The table and images in Fig. 8 confirms the existence of carbon, boron and nitrogen in the sample and the successful synthesis of BNNT powder.

### CNT and BNNT coated membranes characterization results

#### Wettability properties of coated membranes and surface energy

**Figure 9:**
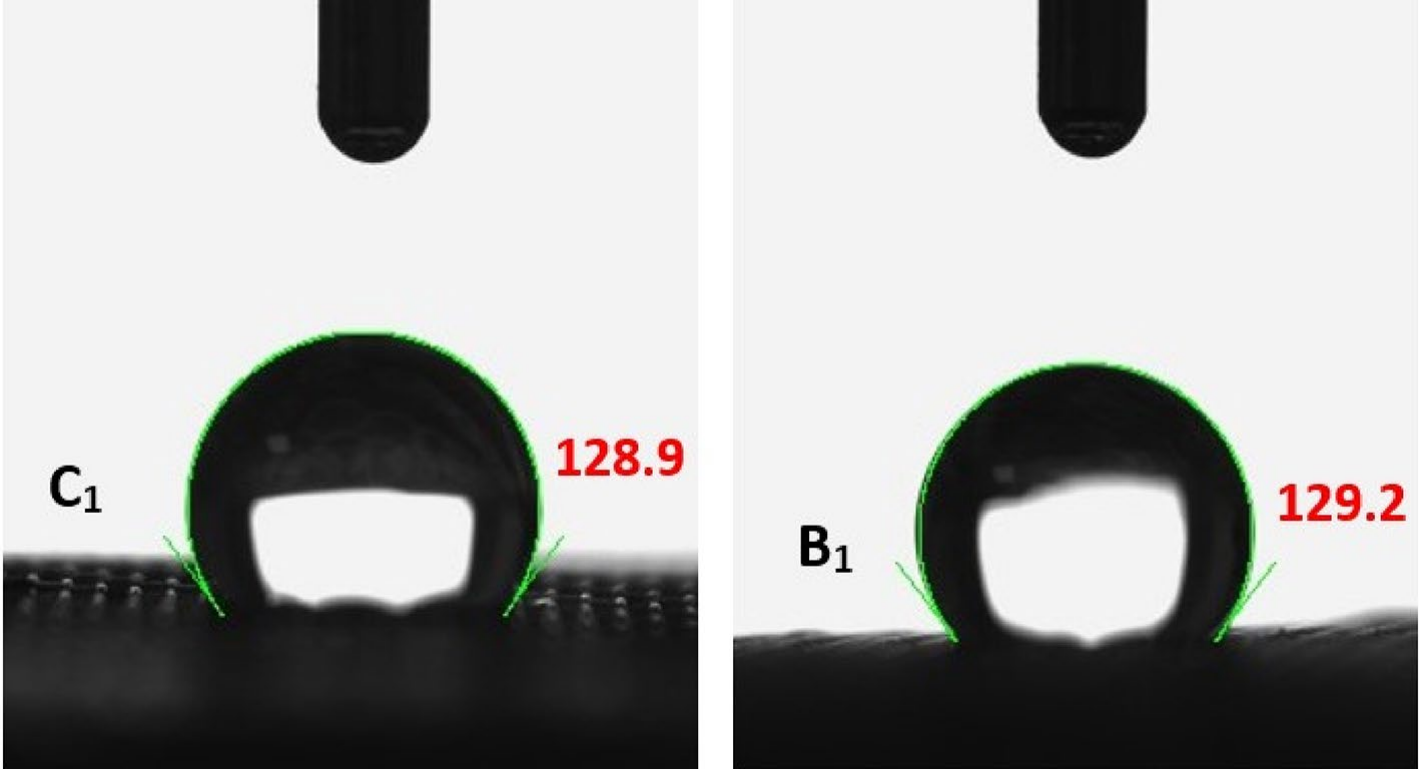
Water contact angle of C_1_ (128.9°) and B_1_ (129.2°) membranes.

**Figure 10:**
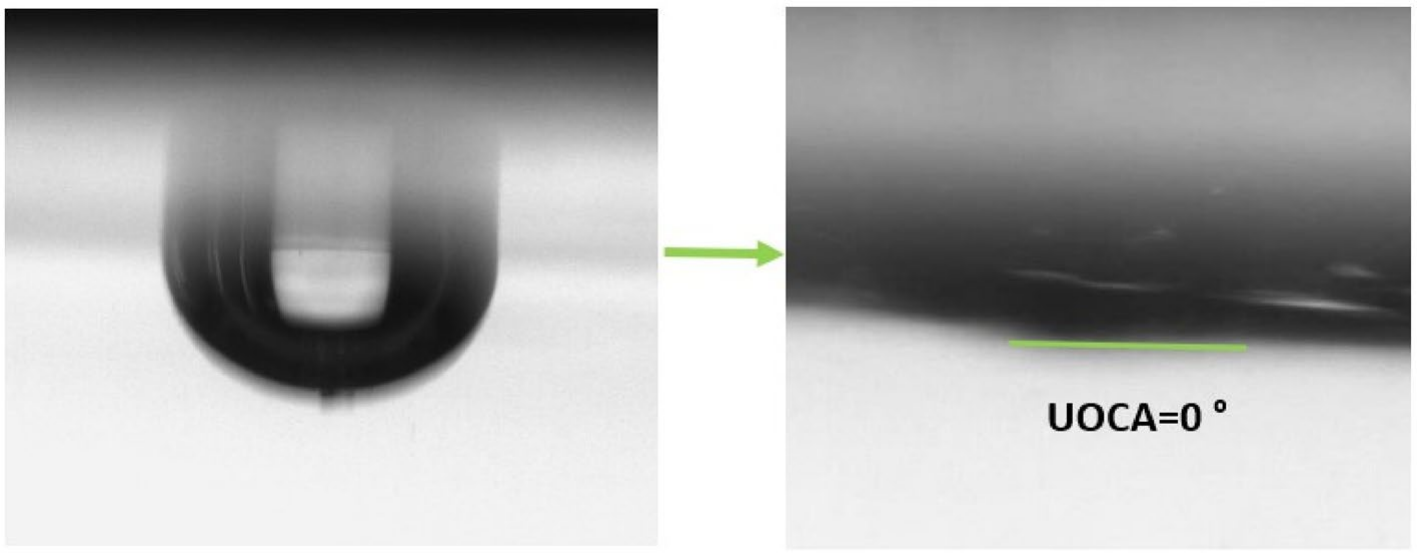
Underwater oil contact angle of both C_1_ and B_1_ membranes (0°).

The wetting properties were studied by measuring static contact angle (WCA, OCA and UOCA) of a 5 µl of water, diiodomethane and decalin respectively to determine the surfaces’ hydrophilicity and oleophilicity. The results are presented in Table 2 which indicates that both C_1_ and B_1_ have hydrophobic properties with the WCA of 128° and 129° respectively. Previous studies also confirm the hydrophobicity of CNT-based membranes with water contact angle of 152°^2^. Figure 9 shows the WCA of C_1_ and B_1_ membranes and Fig. 10 shows the UOCA of them. However, both members with OCA of 0° show superoleophilic behavior in air. Also, underwater OCA was measured and both C_1_ and B_1_ showed superoleophilic behavior with UOCA of 0°. These results were expected because of inherent hydrophobicity property of these nanotubes which stated in the articles^45,46^.

**Table 2:** The results of WCA, OCA and UOCA of prepared C_1_ and B_1_ membranes.

Membrane	WCA (°)	OCA (°)	UOCA (°)
C_1_	128.9	0	0
B_1_	129.2	0	0

Geometric-mean relation^47^ was applied to calculate the C_1_ and B_1_ membranes surface energy according Eqs. (3) and (4) ^47^, because of the relationship between the surface energy and the wettability properties.

3 $$ \cos \theta_{w} = \frac{{2\sqrt {{\upgamma }_{SV}^{d} \,{\upgamma }_{WV}^{d} } + 2\sqrt {{\upgamma }_{SV}^{p} \,{\upgamma }_{WV}^{p} } }}{{{\upgamma }_{WV}^{d} + {\upgamma }_{WV}^{p} }} - 1 $$

4 $$ \cos \theta_{O} = 2\sqrt {\frac{{{\upgamma }_{SV}^{d} }}{{{\upgamma }_{OV}^{d} }}} - 1 $$

Which ${\upgamma }^{{\varvec{p}}}$ states the surface energy of polar (non-dispersive) component and ${\upgamma }^{{\varvec{d}}}$ states the surface energy of dispersive component. $\theta_{w}$ and $\theta_{O}$ denote the water and oil contact angle, respectively and for calculating these items, water and decalin were used in this research. The obtained results are shown in Table 3.

**Table 3:** Surface energy data of the probe liquids used (water and decalin), C_1_ and B_1_.

Component	γ^p^ (mJ/m^2^)	γ^d^ (mJ/m^2^)	γ (mJ/m^2^)
Water	50.8	21.8	72.6
Decalin	0.0	31.5	31.5
C_1_	7.33	50.8	58.13
B_1_	7.87	50.8	58.67

The calculated surface energy of C_1_ (58.13 mJ/m^2^) is equal to this amount for B_1_ (58.67 mJ/m^2^) and they are lower than water’s surface tension (72.6 mJ/m2). This confirms that C_1_ and B_1_ membranes do not tend to get wet with water (Supplementary Information File 1).

#### AFM results of coated membranes

**Figure 11:**
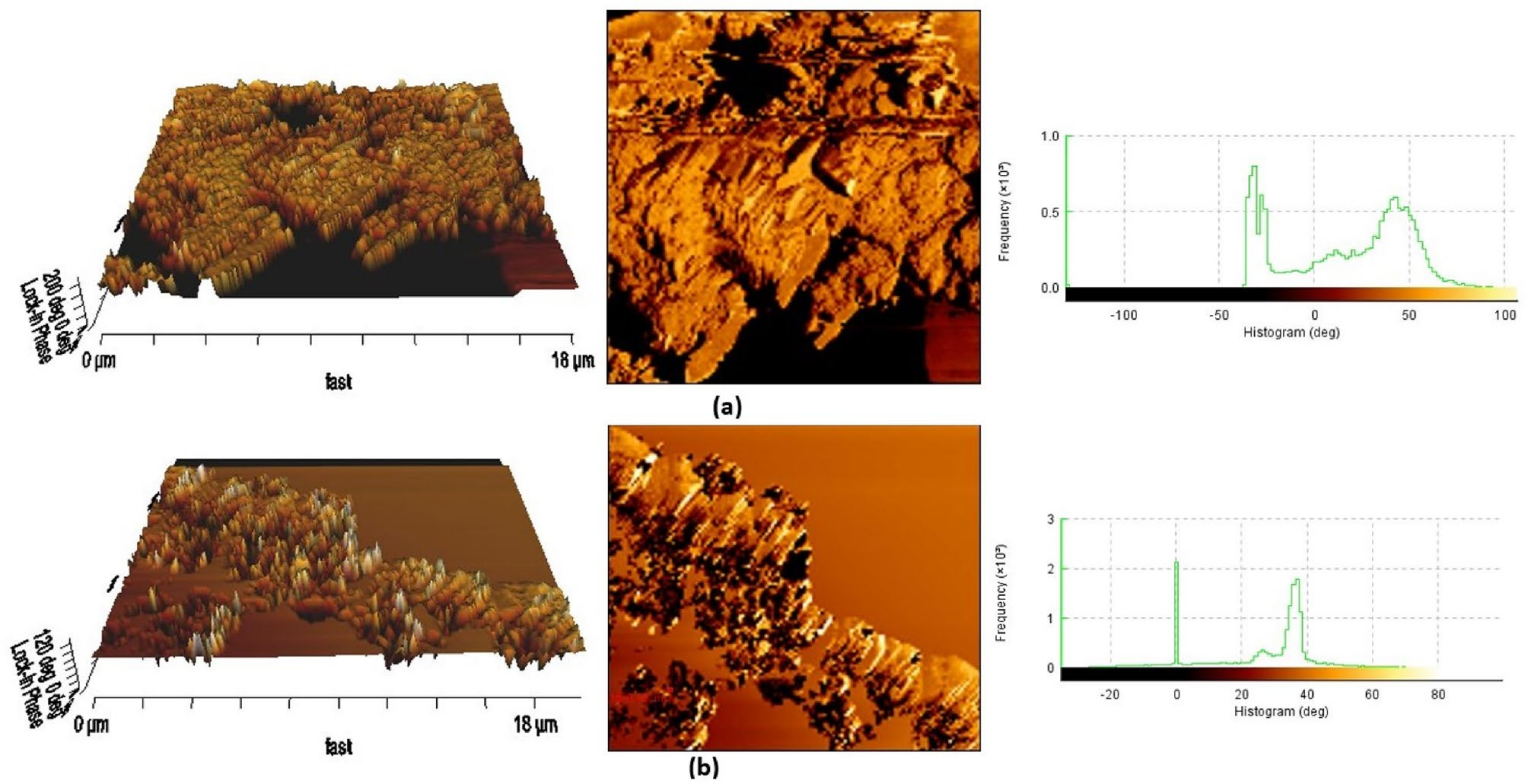
AFM results of (**a**) C_1_ and (**b**) B_1_.

AFM analysis was used to determine the surface topography of both membranes which is indicated in Fig. 11. The Root Mean Square (RMS) was achieved 33.24° for C_1_ and 17.86° for B_1_. The aggregations spread over the coated membrane surface result in a rougher surface, also previous studies showed Ra = 457 nm for multiwalled carbon nanotube coated CA membrane^48^. Determination of the surface roughness by AFM is crucial to the study of particle fouling in nanofiltration^49^. As it is clear from the Fig. 11, the surface roughness of both prepared membranes are very high, so super oleophilic surfaces will be expected. The results obtained from AFM analysis are consistent with the results obtained from the contact angle analysis which mentioned in the previous section.

#### FESEM results of coated membranes

**Figure 12:**
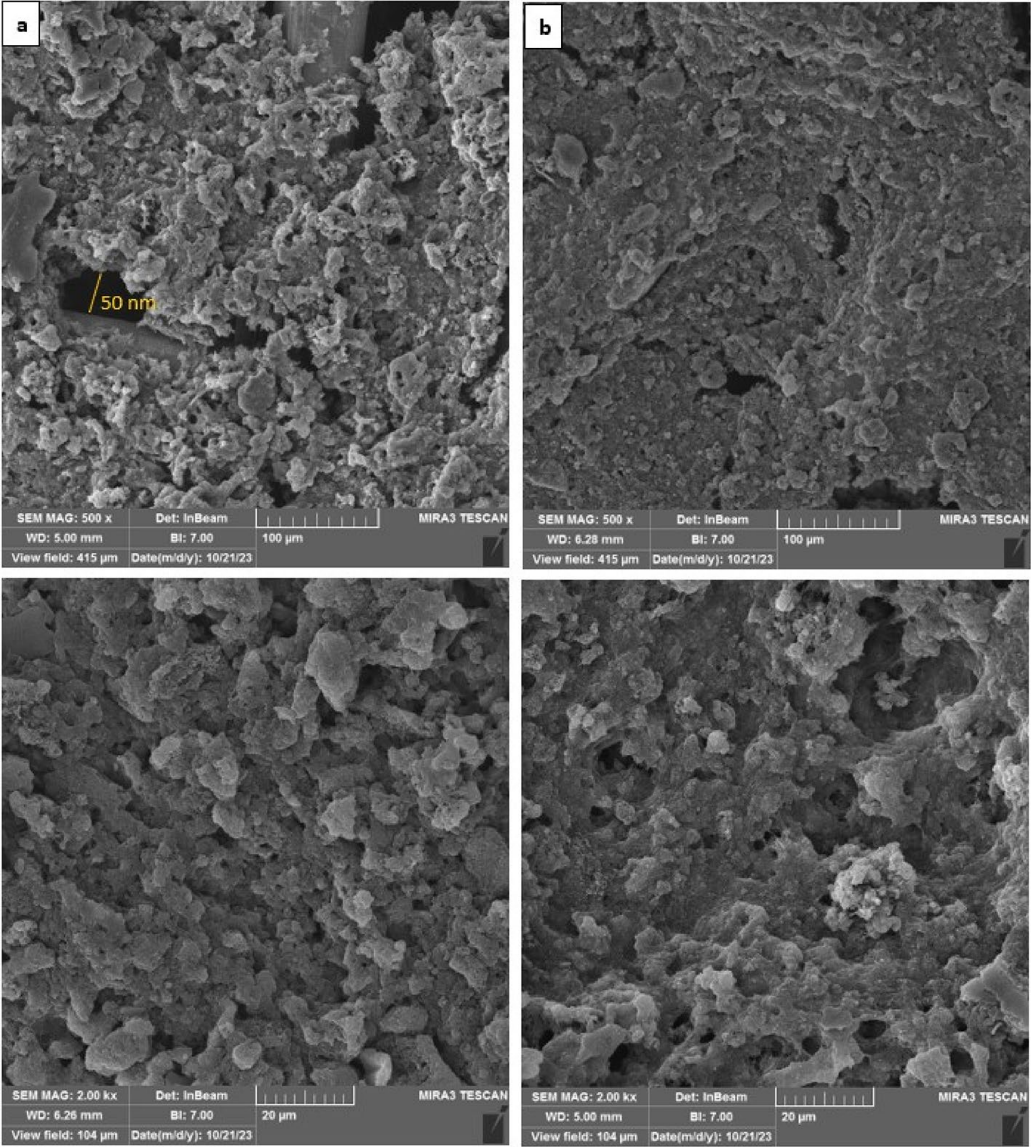
FESEM images of (**a**) C_1_ and (**b**) B_1_ at scale of 100 µm and 20 µm.

The FESEM images of CNT and BNNT coated copper mesh (C_1_ and B_1_) are shown in Fig. 12a,b. The uniform coating of nanoparticles on the wires is well visible, so that the primary copper mesh with an average pore diameter of 125 µm was coated completely by nanotubes coating solution in both C_1_ and B_1_, causing to a considerable reduction in membranes pore diameter to about 50 nm which can greatly facilitate the oil/water separation.

#### Mechanical stability properties of coated membranes

**Figure 13:**
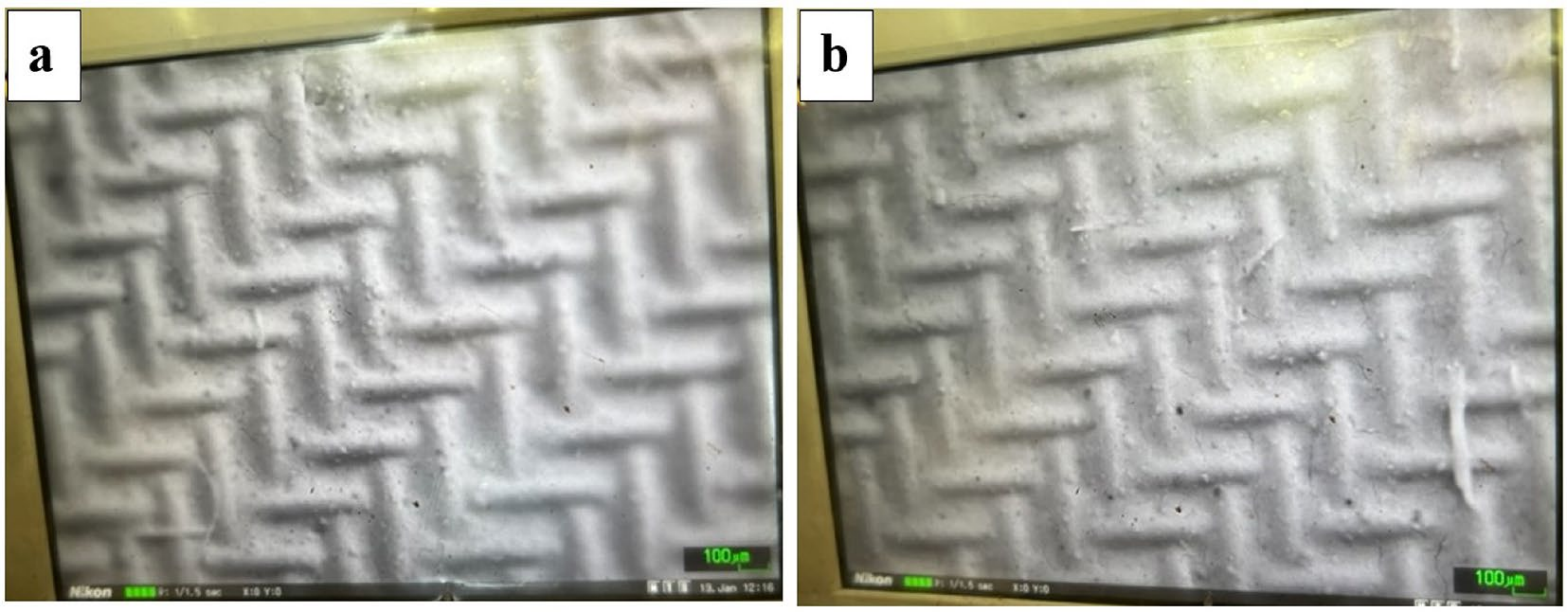
Optical images of (**a**) C_1_ after ultrasonication and (**b**) B_1_ after ultrasonication.

The ultrasonic vibration method was used to investigate mechanical durability. For this purpose, the coated membranes were ultrasonicated for 20 min and after that the membranes were examined for oil/water separation. There was no noticeable change in the water flux and separation efficiency of the membranes after ultrasonication. Also, an optical image to observe the morphology change of the membranes were done. The result shows uniform film formation of CNT and BNNT on the copper mesh like before sonication. Therefore, mechanical durability of the synthesized membranes are confirmed. The optical images of C_1_ and B_1_ membranes after ultrasonication are shown in Fig. 13.

### Oil/water mixture and emulsion separation results

**Figure 14:**
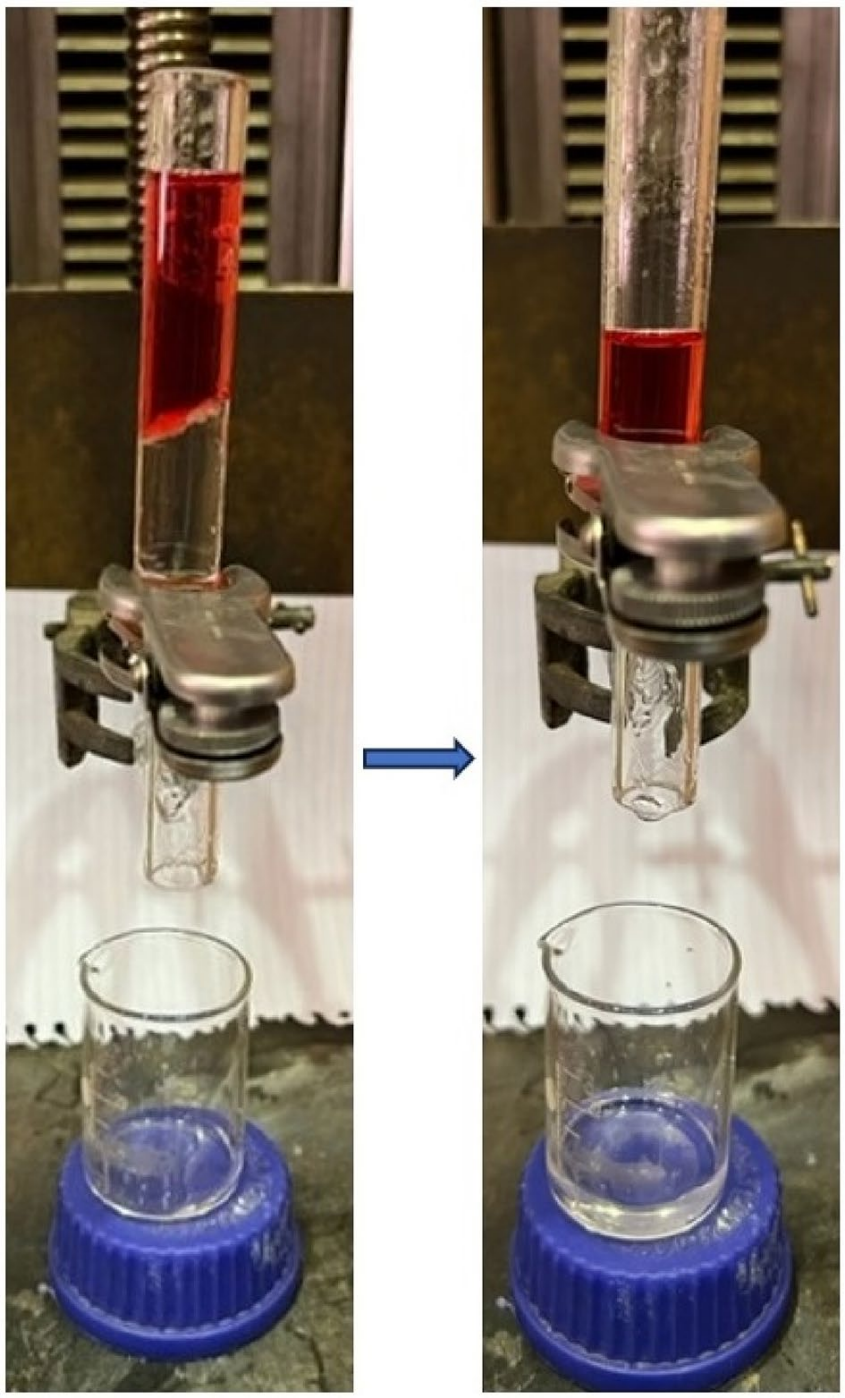
Oil/water mixture separation by coated membranes.

C_1_ and B_1_ membranes were examined to separate free oil/water mixture individually as shown in Fig. 14. The mixture of water (dyed with red color) and decalin (colorless) with a volume ratio of 1:1 was prepared and poured on the membranes which located between two glass tubes in the separation set-up. As it is clear in the figure, the water was retained and decalin passed through both C_1_ and B_1_ prepared membranes. Due to the oleophilicity of the membranes which mentioned in section “wettability properties of coated membranes and surface energy”, this result was expected. The oil flux of C_1_ calculated 458 L m^2^ h^−1^ (Eq. 1) and the separation efficiency was 99% (Eq. 2), while these amounts for B_1_ were 1834 L m^2^ h^−1^ and 99% respectively. Therefore, the amount of flux created by B_1_ has quadrupled. The reason for this increase in flux is the larger internal diameter of boron nitride nanotubes. As mentioned in section “BNNT characterizations results”, the inner diameter of BNNT is about three times larger than CNT used in this study, therefore the extraordinary results of the C_1_ and B_1_ membranes are also a confirmation of this issue.

**Figure 15:**
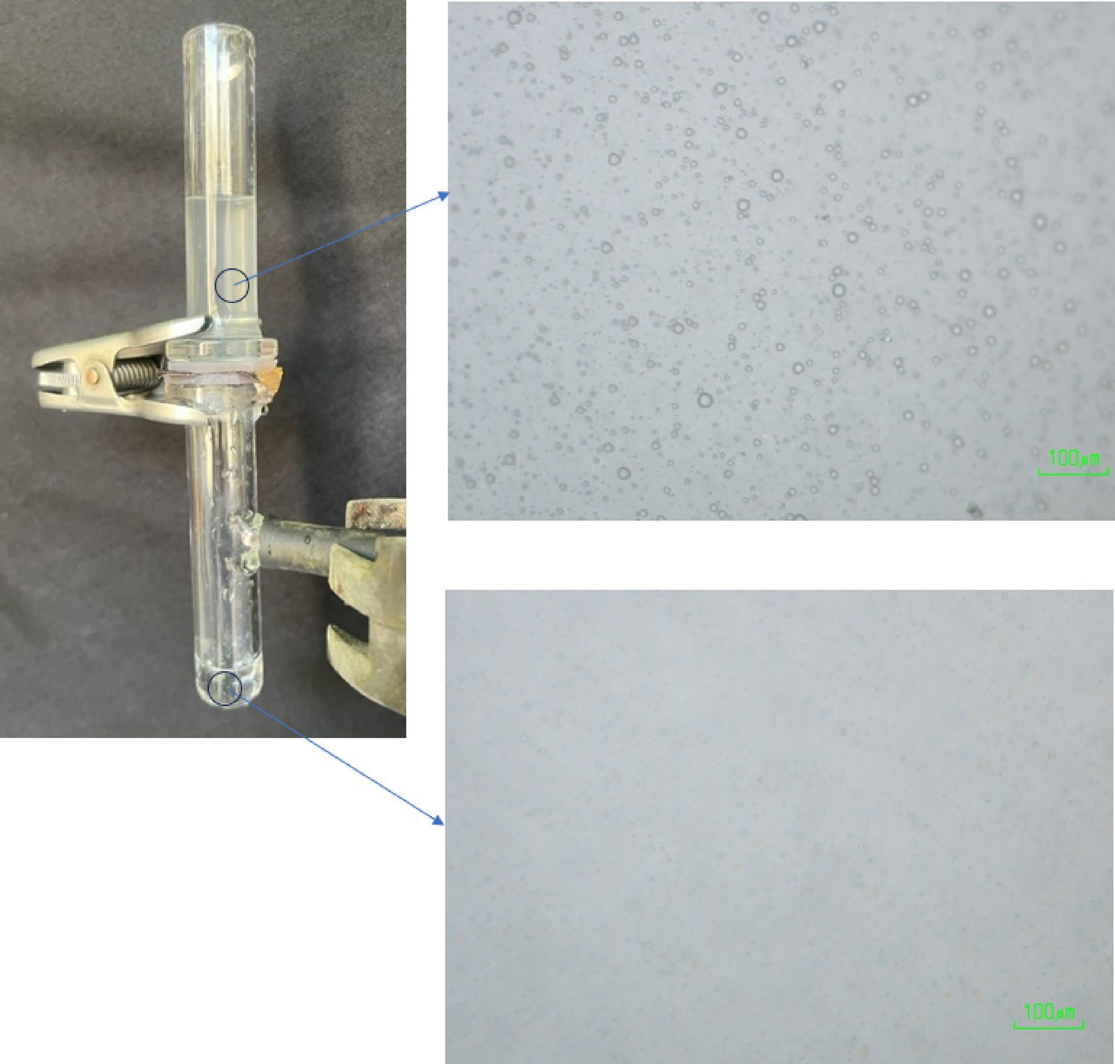
Oil/water emulsion separation by prepared membranes, optical images of emulsion before and after the separation process.

**Figure 16:**
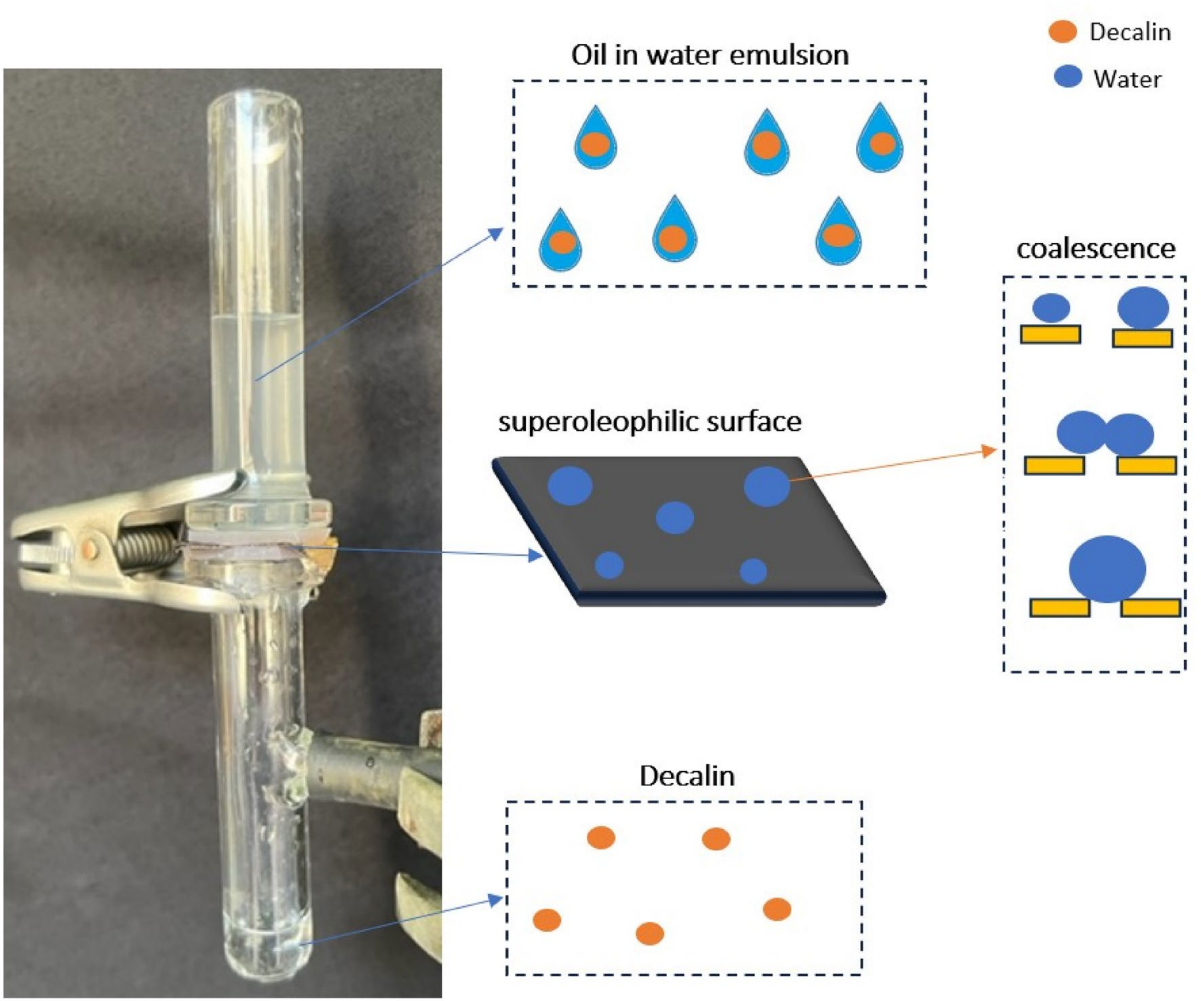
The oil/water emulsion separation mechanism.

In the next step, the membranes were examined to determine their ability to separate oil/water emulsion. The 50 wt% O/W emulsion prepared in the laboratory mentioned in section “Free oil water mixture and oil/water emulsion preparation” poured on the membranes as shown in Fig. 15. Decalin passed through the membranes and water was repelled due to the super oleophilic properties of the prepared membranes. This result was expected because of the oil affinity of the coated membranes. The separation efficiency was about 95% for both of them. The optical images of emulsion before and after the separation process are shown in Fig. 15. It can be clearly seen that the concentration and the size of water drops in the permeate has decreased significantly. The separation mechanism shown in Fig. 16. When the emulsion reached to the coating material on the surface, the oil was passed because of the super-oleophilicity of the surface and water was retentate, then coalescence of the water droplets happened, and the physical mechanism separation was done.

## Conclusion

In this study, after BNNT synthesis and its characteristics assessment, CNT and BNNT coated copper meshes were prepared separately (named C_1_ and B_1_) and the specification of these membranes were investigated. The specific surface area and pore volume was measured 51.6 m^2^ g^−1^, 0.25 cm^3^ g^−1^ respectively which was less than MWCNT used in this study (139 m^2^ g^−1^, 0.84 cm^3^ g^−1^), because of the high purity of the CNT. Several peaks shown in the XRD analysis confirmed the crystalline structure. FESEM test of BNNT powder illustrated the nanostructures of the sample and the inner diameter of that was three times larger than CNT’s inner diameter. The copper mesh with an average pore diameter of 125 µ was coated uniformly by CNT (C_1_) and BNNT (B_1_), causing to a noticeable reduction in membranes pore diameter to about 50 nm which can greatly facilitate the oil/water separation. Wettability properties measurements showed the super-hydrophobicity of the prepared membranes with the WCA of 128° and 129° and the super-oleophilicity with the underwater OCA of 0°. So, in the oil/water separation set-up, after pouring the oil/water mixture, the water was retained and decalin passed through both C_1_ and B_1_ prepared membranes with the flux of 458 and 1834 L m^2^ h^−1^ and the rejection efficiency of 99%. As conclusion, although B_1_ like C_1_ membrane had acceptable ability to separate water and oil, it provides four times higher flux, therefore using BNNT coated membrane is more logical in terms of oil/water separation.

### Supplementary Information

Supplementary Information 1.

Supplementary Information 2.

## Data Availability

The datasets used and analyzed during the current study available from the corresponding author on reasonable request.
