# Supplementary material for: Development of superhydrophobic and superoleophilic CNT and BNNT coated copper meshes for oil/water separation
Source: Sci Rep. 2024 Jun 26;14:14706. doi: 10.1038/s41598-024-65414-5 (PMC11208607; doi:10.1038/s41598-024-65414-5)
Supplement: Supplementary file 1 — Supplementary Information 1. [file 41598_2024_65414_MOESM1_ESM.docx]

**Development of Superhydrophobic and Superoleophilic CNT and BNNT Coated Copper Meshes for Oil/Water Separation**

Fatemeh Hassani^1^, Abdolreza aroujalian^1,^^[[1]](#footnote-1)^*, Alimorad Rashidi^2,^^[[2]](#footnote-2)^**

^1^Faculty of Chemical Engineering, Amirkabir University of Technology (Tehran Polytechnic), Tehran, Iran

^2^Nanotechnology Research Center, Research Institute of Petroleum Industry (RIPI), Tehran, Iran


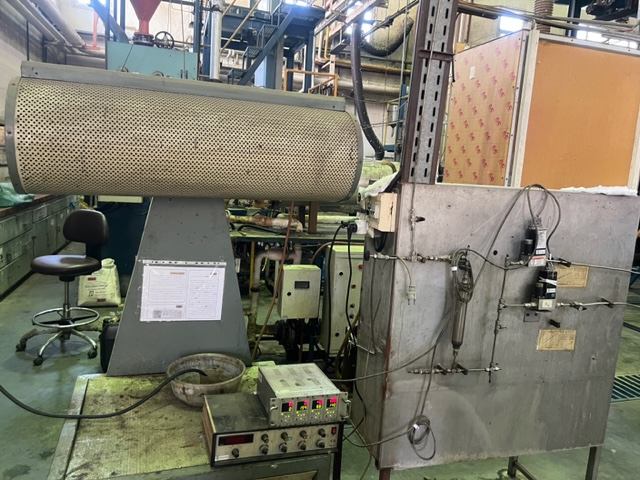


S1. Boron nitride nanotube synthesis by CVD method (actual set-up, tubular furnace)

1. * Corresponding author [↑](#footnote-ref-1)
2. ** Corresponding author

   E-mail addresses: [aroujali@aut.ac.ir](mailto:aroujali@aut.ac.ir) (A. Aroujali), [Rashidiam@ripi.ir](mailto:Rashidiam@ripi.ir) (A. Rashidi). [↑](#footnote-ref-2)
